# Radiographic signs for detection of femoroacetabular impingement and hip dysplasia should be carefully used in patients with osteoarthritis of the hip

**DOI:** 10.1186/1471-2474-15-150

**Published:** 2014-05-08

**Authors:** Ingmar Ipach, Ina-Christine Rondak, Saskia Sachsenmaier, Elisabeth Buck, Roland Syha, Falk Mittag

**Affiliations:** 1 Department of Orthopaedic Surgery, University Hospital of Tuebingen, Hoppe-Seyler-Straße 3, 72074 Tuebingen, Germany; 2 Department of Orthopedic Surgery, Hospital of Ingolstadt, Krumenauerstraße 25, 85021 Ingolstadt, Germany; 3 Department of Medical Statistics and Epidemiology, Technische Universität München, Ismaninger Str. 22, Munich 81675, Germany; 4 Department of Radiology, University Hospital of Tuebingen, Hoppe-Seyler-Straße 3, 72074 Tuebingen, Germany

**Keywords:** Radiographic Signs, Impingement, Hip, Dysplasia, Osteoarthritis

## Abstract

**Background:**

During the last years, terms like acetabular retroversion, excessive overcoverage, and abnormal head-neck-junction with the so called “pistol-grip-deformity” has been added to the classical description of hip dysplasia. These anatomical changes could lead to a femoroacetabular impingement (FAI). Both kinds of FAI has been indentified as a main reason for hip pain and progressive degenerative changes leading to early osteoarthritis of the hip. A lot of radiographic criteria on pelvic views have been established to detect classical dysplasia and FAI. The present study was initiated to assess the hypothesis that age and severity of osteoarthritis affect measurements of different radiographic parameters.

**Methods:**

The pelvic radiographs of 1614 patients were measured for head-ratio, CE-angle, roof obliquity, extrusion-index, depth-to-width ratio, CCD-angle, sharp’s angle. To evaluate the severity of osteoarthritis of the hip the classification by Kellgren and Lawrence was used. Associations between age and radiographic parameters or severity of osteoarthritis were assessed by Spearman’s (ρ) or Kendall’s (r) rank correlation coefficient, respectively.

**Results:**

366 (22.7%) patients presented no sign of osteoarthritis, 367 (22.7%) patients presented I° osteoarthritis, 460 (28.5%) patients presented II° osteoarthritis, 307 (19%) III° osteoarthritis and 114 (7.1%) IV° osteoarthritis of the hip. The mean head-ratio of all patients was 1.13 ± 0.26 (0.76 – 2.40), the mean CE-angle 40.05° ± 10.13° (0° - 70°), the mean roof obliquity was 35.27°± 4.96° (10° – 55°), the mean extrusion-index was 12.99 ± 9.21 (6.20 – 95.2), the mean depth-to-width ratio was 59.30 ± 8.90 (6.30 – 100), the mean CCD-angle was 127.68° ± 7.22° (123° – 162°) and the mean sharp’s angle was 9.75° ± 5.40° (1° - 34°) There was a weak association between age and the severity of osteoarthritis of the hips (left: r = 0.291; right: r = 0.275; both P < 0.001) with higher osteoarthritis levels observable for elderly patients).

**Conclusion:**

Severity of osteoarthritis has a negative impact on measurements of different radiographic parameters. Therefore - in our opinion - epidemiological studies on prearthrotic deformities should only be performed in healthy adults with no signs of osteoarthritic changes.

## Background

During the last years, terms like acetabular retroversion, excessive overcoverage, and abnormal head-neck-junction with the so called “pistol-grip-deformity” [1-4] has been added to the classical description of hip dysplasia with a lateral CEA-angle of less then 25° and a roof obliquity angle of more than 10° [5]. These anatomical changes could lead to a femoroacetabular impingement (FAI). Two kinds of FAI have been described. In Cam impingement a repetitive contact between an abnormal head-neck-junction and the acetabular rim causes cartilage damage in the anterosuperior area of the acetabulum. In Pincer impingement a direct contact between the femoral neck and a local/generalized overcovered acetabulum leads also to repetitive damage of the cartilage at the acetabular rim [6-16].

Both kinds of FAI has been indentified as a main reason for hip pain and progressive degenerative changes leading to early osteoarthritis of the hip [1-4,6,7].

The diagnosis of FAI should be based on detailed physical examination and appropriate imaging studies. A lot of radiographic criteria on pelvic views have been established to detect classical dysplasia and FAI [17-21]. The head-ratio has already described as reliable for the detection of “pistol-grip-deformity” [7,8,22]. It has also been shown that radiographic FAI findings are very common in a population of healthy young adults [23].

Goodman et al. have shown that “pistol-grip-deformity” is due to a three-dimensional structural abnormality with no change in severity with age [24].

According to Kellgren and Lawrence [25] progressive degenerative changes lead to osteophytes, narrowing of the joint space, and deformity of the bone ends. These changes may have a negative impact on radiographic parameters. The present study was initiated to assess the influence of severity of osteoarthritis on radiographic parameters.

## Methods

Ethical approval has been received by the ethic committee of Tübingen (025/2014R). We analysed our data bank for all pelvic-views which has been performed in our institution in the period between 1^st^ January 2006 and 31^st^ December 2011.

To exclude the negative influence of pelvic tilt and rotation on radiographic parameters, only standardized pelvic radiographs were included in this study. The distance between the tip of the coccyx and the middle of the symphysis was 32 mm for men and 47 mm for women and the teardrop sign appeared symmetrical [1].

The pelvic-views were measured for head-ratio, CE-angle, roof obliquity, extrusion-index, depth-to-width ratio, CCD-angle, sharp’s angle [8,17-20,22,26]. In cases with the presents of a THA, fracture or Dysplasia Crowne II/IV on one side, only the other was measured.

To evaluate the severity of osteoarthritis of the hip the classification by Kellgren and Lawrence was used [25].

### Statistical analysis

Statistical analyses were conducted with the use of IBM SPSS Statistics 21 and R software, version 3.0.0 (R Core Team).

In order to account for repeated measurements of both hips on patient level, analysis was conducted separately for left and right hips or using summarized values for both sides (i.e. mean or maximum values of both hips).

When analyzing the influence of age on severity of osteoarthritis different radiographic parameters, left and right hip were analyzed separately.

When analyzing the influence of severity of osteoarthritis on the different radiographic parameters, the most severe hip and corresponding measurements or in case of equal severity on both sides, averaged measurements over both hips were considered for each patient.

Categorical variables are presented as frequencies, percentages, and continuous variables as means and standard deviations, or medians and interquartile ranges (Box-plots) for variables with skewed distributions.

Associations between age and radiographic parameters or severity of osteoarthritis were assessed by Spearman’s (ρ) or Kendall’s (τ) rank correlation coefficient, respectively. Group comparisons based on severity of osteoarthritis of continuous measurements were conducted using either the one-way analysis of variances (ANOVA) or the Kruskall-Wallis test, as appropriate.

All reported P values are two-tailed, with a P value of 0.05 indicating statistical significance and have not been adjusted for multiple testing.

## Results

The pelvic radiographs of 1614 Patients were included into this study. In 1052 of all cases both sides were measured in 562 cases only the left (n = 311) or right side (n = 251) were measured for radiographic signs of FAI and dysplasia. The mean age of all patients was 60.2 years ± 17.1. 44.4% of all patients were male, 55.6% were female.

366 (22.7%) patients presented no sign of osteoarthritis, 367 (22.7%) patients presented I° osteoarthritis, 460 (28.5%) patients presented II° osteoarthritis, 307 (19%) III° osteoarthritis and 114 (7.1%) patients presented IV° osteoarthritis of the hip.

**Figure 1:**
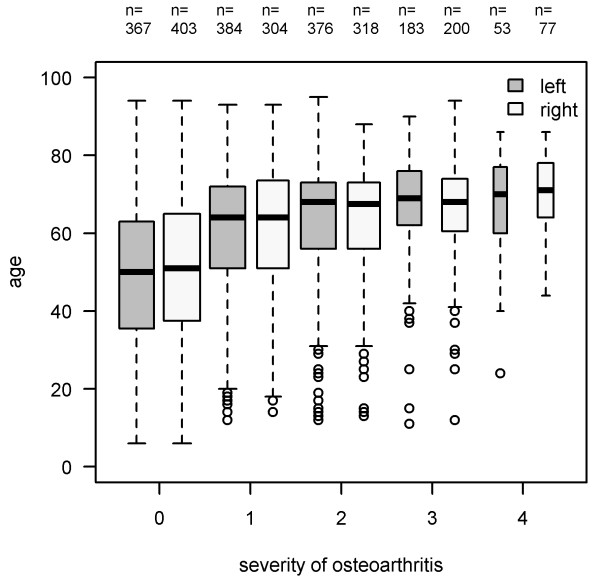
**Association between severity of osteoarthritis and age.** Y-axis: age in years, X-axis: severity of osteoarthritis according to Kellgren-Lawrence (Kendall’s correlation coefficient for left hips: r = 0.291; and right hips: r = 0.275; both p < 0.001).

The mean values of all different radiographic parameters are demonstrated in Table 1. There was a weak association between age and the severity of osteoarthritis of the hips (left: τ = 0.291; right: τ = 0.275; both P < 0.001) with higher osteoarthritis levels observable for elderly patients (Figure 1).

**Table 1:** Measurement values for the different radiographic parameters of both hips

**n=**	**Sharps-angle (in degree)**	**Head-ratio**	**CE-angle (in degree)**	**Roof obliquity (in degree)**	**Extrusion-index (in %)**	**Depth-to-width ratio (in %)**	**CCD-angle (in degree)**
1614	9.75° ± 5.40° (1° - 34°)	1.13 ± 0.26 (0.76 – 2.40)	40.05° ± 10.13° (0° - 70°)	35.27 ± 4.96 (10 – 55)	12.99 ± 9.21 (6.20 – 95.2)	59.30 ± 8.90 (6.30 – 100)	127.68° ± 7.22° (123° – 162°)

The results are presented as means and standard deviations (Minimum – Maximum).

**Figure 2:**
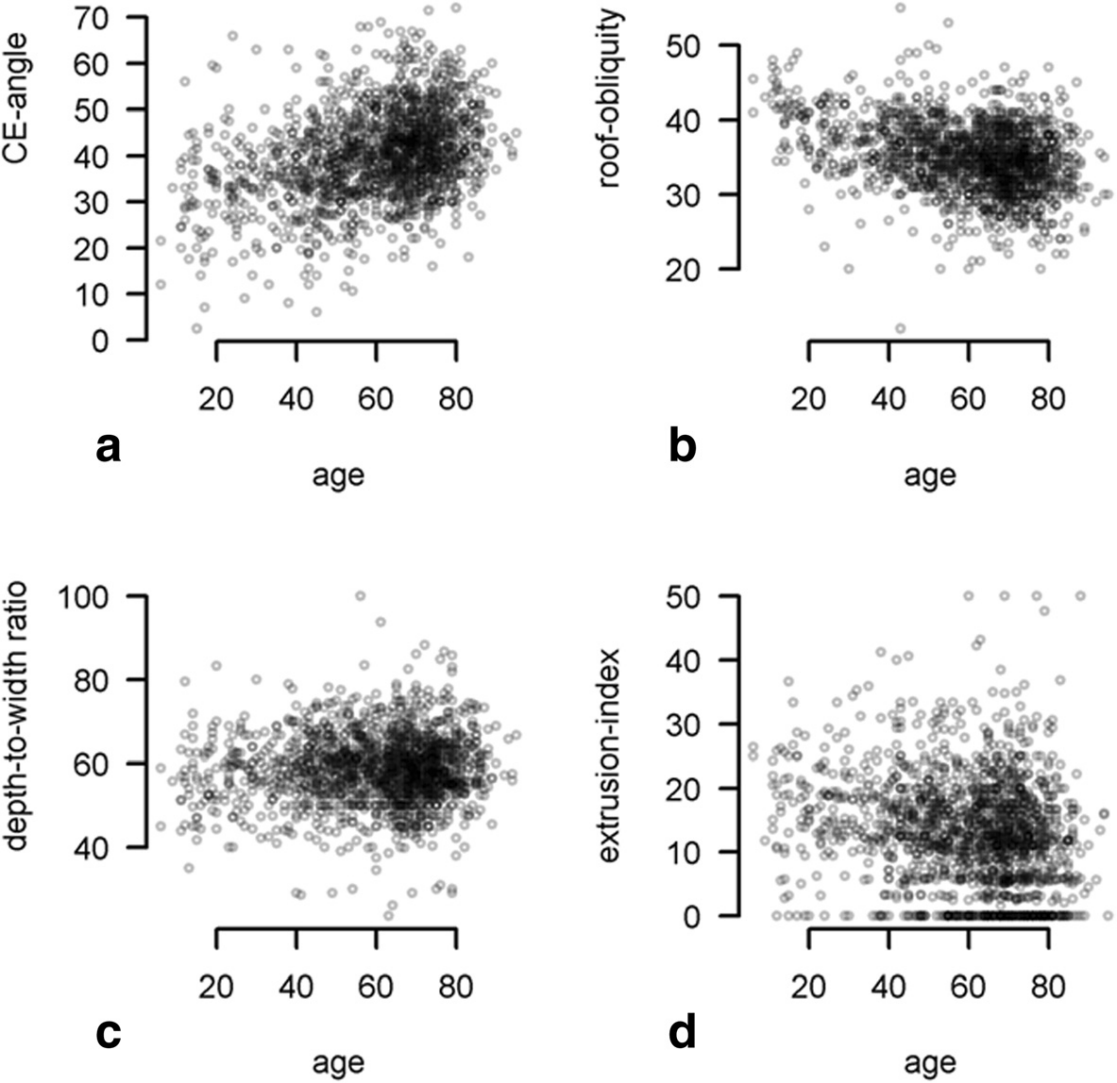
**Correlation between CE-angle, roof-obliquity, depth-to-width-ratio, extrusion-index and age. a**: Correlation between age and CE-angle. X-axis: age in years, Y-axis: CE-angle in degree. There was a weak monotone correlation between age and CE-angle (ρ = 0.334). **b**: Correlation between age and roof-obiquity. X-axis: age in years, Y-axis: roof obliquity in degree. There no correlation between age and roof obliquity (ρ = -0.133). **c**: Correlation between age and depth-to-width-ratio. X-axis: age in years, Y-axis: depth-to-width-ratio in %. There was no correlation between age and depth-to-width-ratio (ρ = 0.033) **d**: Correlation between age and extrusion-index. X-axis: age in years, Y-axis: extrusion-index in %. There was weak negative correlation between age and extrusion-index (ρ = -0.218).

**Figure 3:**
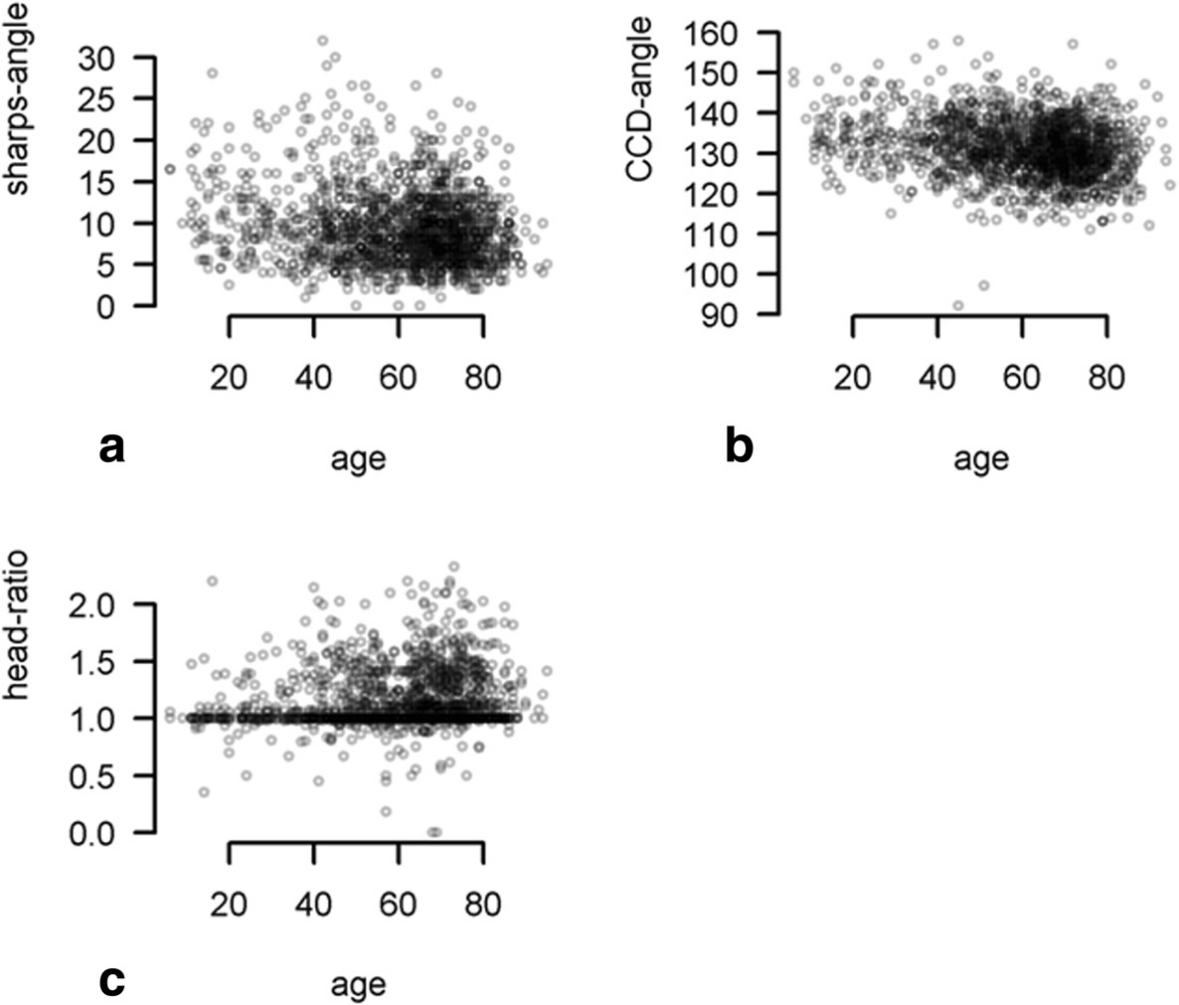
**Correlation between sharps-angle, CCD-angle, head-ratio and age. a**: Correlation between age and sharps-angle. X-axis: age in years, Y-axis: sharps-angle in degree. There was weak negative correlation between age and sharps-angle (ρ = -0.299) **b**: Correlation between age and CCD-angle. X-axis: age in years, Y-axis: CCD-angle in degree. There was weak negative correlation between age and CCD-angle (ρ = -0.205) **c**: Correlation between age and head-ratio. X-axis: age in years, Y-axis: head-ratio There was no correlation between age and head-ratio (ρ = 0.135).

**Figure 4:**
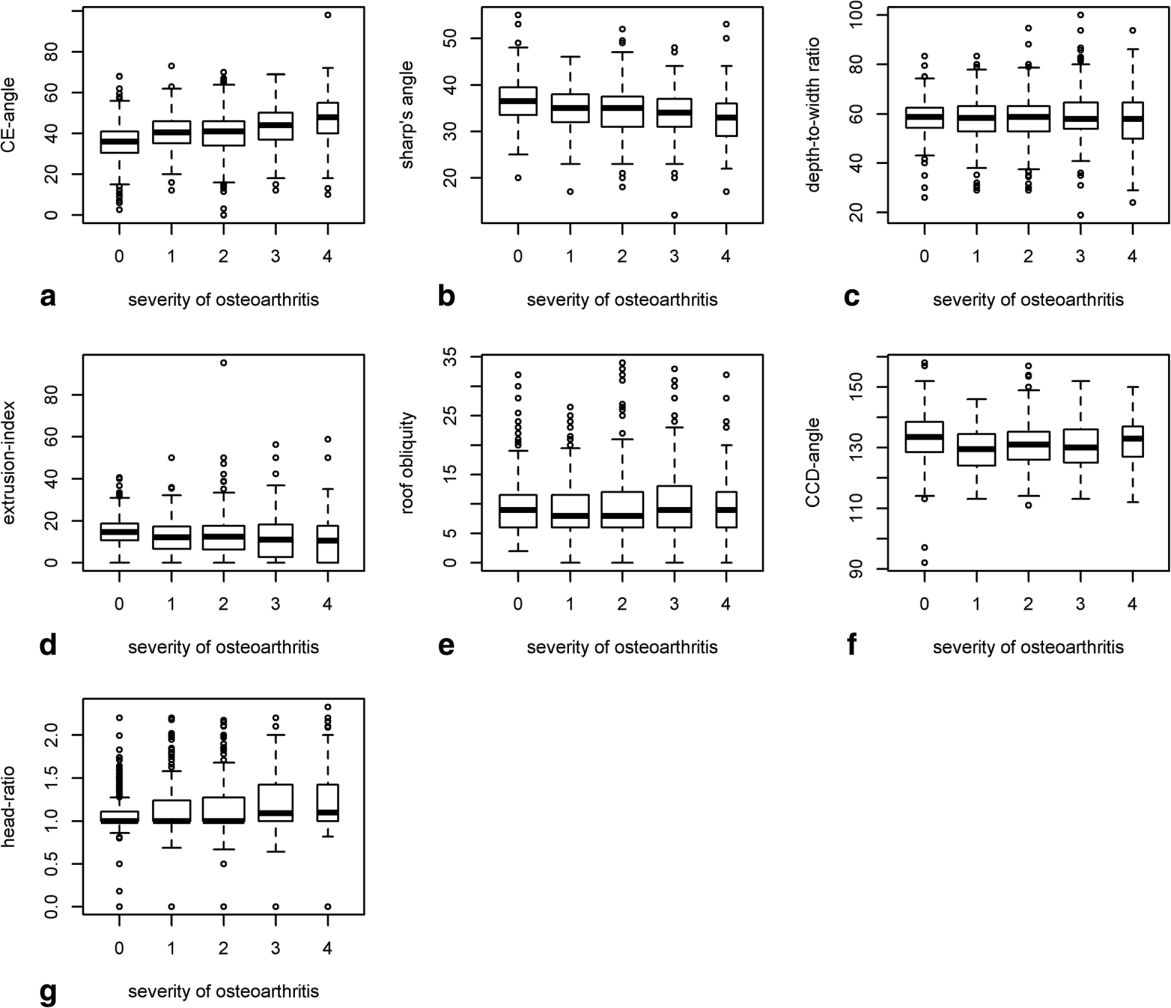
**Association between severity of osteoarthritis and radiographic parameters. a**-**g**: Patient based measurement values of CE-angle, sharps-angle, Extrusion-index, depth-to-width ration, CCD-angle, head-ratio and roof-oblique by severity of osteoarthritis of the hip (Kellgren-Lawrence 0-4) [most severe hip and corresponding measurements or in case of equal severity on both sides, averaged measurements over both hips were considered]. **a**: X-axis: group 0-4 according to severity of osteoarthritis, Y-axis: CE-angle in degree. There was a statistical significant difference in the CE-angle between the groups (p < 0.001). **b**: X-axis: group 0-4 according to severity of osteoarthritis Y-axis: sharp’s angle in degree. There was a statistical significant difference in the sharp’s angle between the groups (p < 0.001) **c**: X-axis: group 0-4 according to severity of osteoarthritis Y-axis: depth-to-width-ratio in%. No statistical significant difference between the groups was seen (p = 0.535). **d**: X-axis: group 0-4 according to severity of osteoarthritis Y-axis: extrusion-index in%. There was a statistical significant difference in the extrusion-index between the groups (p < 0.001) **e**: X-axis: group 0-4 according to severity of osteoarthritis, Y-axis: roof-obliquity in degree. No statistical significant difference between the groups was seen (p = 0.18). **f**: X-axis: group 0-4 according to severity of osteoarthritis Y-axis: CCD-angle in degree. There was a statistical significant difference in the CCD-angle between the groups (p < 0.001). **g**: X-axis: group 0-4 according to severity of osteoarthritis Y-axis: head-ratio. There was a statistical significant difference in the head-ratio between the groups (p < 0.001).

The correlation between age and head-ratio, CE-angle, roof obliquity, extrusion-index, depth-to-width ratio, CCD-angle, sharp’s angle is demonstrated in Figures 2 and 3. Weak monotone associations were also observed between age and mean CE-angle (ρ = 0.334) (p < 0.001), mean sharps angle (ρ = -0.299) (p < 0.001), mean extrusion-index (ρ = -0.218) (p < 0.001), and mean CCD-angle (ρ = -0.205) (p < 0.001). On the other hand no correlations were seen between age and depth-to-width ratio (ρ = 0.033) (p < 0.181), roof obliquity (ρ = 0.133) (p < 0.001) and head-ratio (ρ = 0.135) (p < 0.001). These results imply that age has only a weak or no impact on the different radiographic parameters for FAI and dysplasia. One-way ANOVA revealed statistical significant differences between patient groups based on severity (Kellgren-Lawrence 1°-4°) of osteoarthritis for the following measurements: CE-angle (p < 0.001), sharps-angle (p < 0.001), Extrusion-index (p < 0.001), and CCD-angle (p < 0.001). No statistical significant difference between patient groups based on severity (Kellgren-Lawrence 1°-4°) and depth-to-width ratio was seen (p = 0.535). Assuming a skewed distribution of head-ratio and roof-obliquity measurements, the nonparametric Kruskall-Wallis-test revealed a statistical significant difference between osteoarthritis groups head-ratio (P < 0.001) but none for roof-obliquity (Figure 4) (P = 0.18). These results imply that there is an association between the different radiographic parameters (excluded roof-obliquity and depth-to-width ratio) and the severity of osteoarthritis of the hip.

## Discussion

During the last years a lot of studies have shown changes in the acetabular geometry and the head neck region as a main reason for an early osteoarthritis of the hip. Terms like femoroacetabular impingement have become more and more important in the explanation for hip pain and the early development of OA. These morphologic changes in the acetabular geometry such as an excessive overcoverage or an acetabular retroversion or changes in the head-neck-region lead to the so-called Pincer- or Cam-impingement [6-18].

However, the “classic” definition of hip-dysplasia with an undercoverage of the femoral head (reduced CE-angle) or a steep acetabular roof (increased roof obliquity) is still playing an important role in the indication of total hip arthroplasty in young adults [26-30].

While watching different epidemiological studies, an abnormal hip morphology with acetabular dysplasia has been reported in about 51- 80% of all cases with OA of the hip [23]. Including radiographic findings for an excessive overcoverage of the femoral head, acetabular retroversion or an abnormal head-neck-junction, as a dysplastic change, hip-dysplasia was seen in nearly 97% of all patients [26].

The prevalence of acetabular dysplasia has been reported to be about 3.8% in the British population [28] and 4.5% in Chinese men [29]. An abnormal head-neck-junction seems to be present in about 40%-50% of all hips with OA [26,30]. A recent study has shown a high prevalence of radiographic finding for FAI in a cohort of 2081 healthy adults. The prevalence of CAM-impingement was up to 40%, the prevalence of Pincer-impingement up to 50% [23].

The present study was performed to assess the impact of severity of osteoarthritis on different radiographic measurement values. We were able to show that degenerative changes has an negative impact on radiographic parameters for FAI and hip dysplasia. To our knowledge this was the first study focusing on this topic.

The posterior head tilt in osteoarthritic hips has been discussed as being an acquired deformity created by the formation of osteophytes [8]. The present study support these findings, as the head-ratio demonstrated an increase with the severity of osteoarthritis. On the other hand it was shown that this deformity did not change with age and therefore it could be seen as a prearthrotic deformity and not as secondary to degeneration [24].

We not several limitations in this study. A testing on intra- and interobserver reliability hasn’t be performed in this study, but previous studies already focussed on this topic [31]. Clohisy et al. [32] and Gosving et al. [33] reported about poor results for inter- and intra-observer reliability of measurements of radiographic signs for acetabular dysplasia, head-neck offset and pelvic tilt. Other studies were able to show a high inter- and intraobserver reliability for different radiographic parameters by using the Balnd-Altman-method [26,31,34,35]. The discrepancy between these studies could be explained by the using of different statistical methods. The kappa-coefficient by Cohen [34] is the first choice for testing inter- and intraobserver quality of categorical variables, it should not be used for data on a continuous level [34,35]. Correlation coefficients for testing reliability between two observers should not be used at all [34]. Therefore the Bland-Altman-method is a better choice for assessing agreement of continuous data [34,35].

Nevertheless the inter- and intrabserver quality of radiographic findings in the diagnostic of dysplasia, FAI and excessive overcoverage is one of the main problem in the clinical routine. Therefore the use of a three-dimensional imaging might be helpful in unclear cases.

## Conclusion

There seems to be a negative impact of severity of osteoarthritis on different radiographic parameters for the detection of femoroacetabular impingement and hip dysplasia. Therefore - in our opinion - epidemiological studies on prearthrotic deformities and radiographic paramters for FAI and hip dysplasia should only be performed in adults with no signs of osteoarthritic changes.

## Abbreviations

CE-angle: Centre-edge-angle; CCD-angle: Caput-collum-diaphysis-angle; FAI: Femoroacetabular impingement; OA: Osteoarthritis; THA: Total hip arthroplasty.

## Competing interests

The authors declare that they have no competing interests.

## Authors’ contributions

II: Drafting the manuscript IR: statistical analysis SS: graphical design, recruiting x-rays EB: performing measurements RS: controlling accuracy of measurements and quality of x-rays FM: Study design. All authors read and approved the final manuscript.

## Pre-publication history

The pre-publication history for this paper can be accessed here:

http://www.biomedcentral.com/1471-2474/15/150/prepub

## References

[B1] Beall DP Sweet CF Martin HD Imaging findings of femoroacetabular impingement syndrome Skeletal Radiol 2005 34 91 701 10.1007/s00256-005-0932-9 16172860

[B2] James SL Ali K Malara F Young D O’Donnell J Connell DA MRI findings of femoroacetabular impingement AJR 2006 187 1412 1419 10.2214/AJR.05.1415 17114529

[B3] Tannast M Kubiak-Langer M Langlotz F Puls M Murphy SB Siebenrock KA Non invasive threedimensional assessment of femoroacetabular impingement J Orthop Res 2007 25 122 131 10.1002/jor.20309 17054112

[B4] Siebenrock KA Schoeninger R Ganz R Anterior femoro-acetabular impingement due to acetabular retroversion: treatment with periacetabular osteotomy J Bone Joint Surg Am 2003 5 A 278 286 12571306 10.2106/00004623-200302000-00015

[B5] Miller MD Review of Orthopaedics 5th edition 2008 Philadelphia: Saunders Elsevier

[B6] Ganz R Parvizi J Beck M Leunig M Nötzli H Siebenrock KA Femoroacetabular impingement: a cause for osteoarthritis of the hip Clin Orthop Relat Res 2003 417 112 120 14646708 10.1097/01.blo.0000096804.78689.c2

[B7] Nötzli HP Wyss TF Stoecklin CH Schmid MR Treiber K Hodler J The contour of the femoral head-neck junction as a predictor for the risk of anterior impingement J Bone Joint Surg (Br) 2002 84 556 560 10.1302/0301-620X.84B4.12014 12043778

[B8] Murray RO The aetiology of primary osteoarthritis of the hip Br J Radiol 1965 38 810 824 10.1259/0007-1285-38-455-810 5842578

[B9] Jäger M Wild A Westhoff B Femoroacetabular impingement caused by a femoral osseous head-neck bump deformity: clinical, radiological, and experimental results J Orthop Sci 2004 9 256 263 10.1007/s00776-004-0770-y 15168180

[B10] Koegh MJ Batt ME A review of femoroacetabular impingement in athletes Sports Med 2008 38 863 878 10.2165/00007256-200838100-00005 18803437

[B11] Stafford G Witt J The anatomy, diagnosis and pathology of femoroacetabular impingement Br J Hosp Med 2009 70 72 77 10.12968/hmed.2009.70.2.38904 19229146

[B12] Anda S Terjesen T Kvistad KA Svenningsen S Acetabular angles and femoral anteversion in dysplastic hips in adults: CT-investigation J Comput Assist Tomogr 1991 15 1 115 120 10.1097/00004728-199101000-00018 1987179

[B13] Anderson LA Peters CL Park BB Stoddard GJ Erickson JA Crim JR Acetabular cartilage delamination in femoroacetabular impingement. Risk factors and Magnetic Resonance imaging diagnosis J Bone Joint Surg Am 2009 91 305 313 10.2106/JBJS.G.01198 19181974

[B14] Fadul DA Carrino JA Imaging of femoroacetabular impingement J Bone Joint Surg Am 2009 91 138 143 10.2106/JBJS.H.01449 19182042

[B15] Murphy SB Ganz R Müller ME The prognosis in untreated dysplasia of the hip. A study of radiographic factors that predict the outcome J Bone Joint Surg Am 1995 77 7 985 989 7608241 10.2106/00004623-199507000-00002

[B16] Laude F Boyer T Nogier A Anterior femoroacetabular impingement Joint Bone Spine 2007 74 127 132 10.1016/j.jbspin.2007.01.001 17337228

[B17] Lequesne M Coxometry measurement of the basic angles of the adult radiographic hip by a combined protractor Rev Rhum Mal Osteoartic 1963 30 479 485 14088029

[B18] Tannast M Siebenrock KA Anderson SE Femoroacetabular Impingement: radiographic diagnosis – what the radiologist should know Am J Roentgenol 2007 188 6 1540 1552 10.2214/AJR.06.0921 17515374

[B19] Steppacher SD Tannast M Werlen S Siebenrock KA Femoral morphology differs between deficient and excessive acetabular coverage Clin Orthop Relat Res 2008 466 4 782 790 10.1007/s11999-008-0141-7 18288550 PMC2504673

[B20] Tönnis D Heinecke A Acetabular and femoral anteversion: relationship with osteoarthritis of the hip J Bone Joint Surg Am 1999 81 12 1747 1770 10608388 10.2106/00004623-199912000-00014

[B21] Wiberg G Studies on dysplastic acetabula and congenital subluxation of the hip joint: With special reference to the complications of osteoarthritis Acta Chir Scan 1939 58 7 38

[B22] Ipach I Mittag F Sachsenmaier S Heinrich P Kluba T A new classification for “pistol grip deformity”- correlation between the severity of the deformity and the grade of osteoarthritis of the hip Fortschr Röntgenstrahl 2010 183 4 365 371 10.1055/s-0029-1245817 21080301

[B23] Laborie LB Lehmann TG Engeæter I Eastwood DM Engeæter LB Rosendahl K Prevalence or radiographic findings thought to be associated with femoroacetabular impingement in a population-based cohort of 2081 healthy young adults Radiology 2011 260 494 502 10.1148/radiol.11102354 21613440

[B24] Goodman DA Feighan JE Smith AD Latimer B Buly RL Cooperman DR Subclinical slipped capital femoral epiphysis. Relationship to osteoarthrosis of the hip J Bone Joint Surg Am 1997 79 10 1489 1497 9378734 10.2106/00004623-199710000-00005

[B25] Kellgren JH Lawrence JS Radiological assessment of osteo-arthrosis Ann Rheum Dis 1957 16 494 502 10.1136/ard.16.4.494 13498604 PMC1006995

[B26] Ipach I Mittag F Syha R Kunze B Wolf P Kluba T Indications for total hip arthroplasty in young adults - idiopathic osteoarthritis seems to be overestimated Röfo 2012 184 3 239 247 22274871 10.1055/s-0031-1299052

[B27] Harris WH Etiology of osteoarthritis of the hip Clin Orthop 1986 213 20 33 3780093

[B28] Cooperman DR Wallensten R Stulberg SD Post-reduction avascular necrosis in congenital dislocation of the hip J Bone Joint Surg Am 1980 62 2 247 258 7358756

[B29] Lau EM Lin F Lam D Silman A Croft P Hip osteoarthritis and dysplasia in Chinese men Ann Rheum Dis 1995 54 12 965 969 10.1136/ard.54.12.965 8546528 PMC1010061

[B30] Stulberg SD Cordell LD MacEwen GD Amstutz HC Unrecognized childhood hip disease: a major cause of idiopathic osteoarthritis of the hip The Hip Procs Third Open Scientific Meeting of the Hip Society St Louis 2010 St. Louis: C V Mosby 212 218

[B31] Ipach I Arlt EM Mittag F Kunze B Wolf P Kluba T A classification-system improves the intra- and interobserver reliability of radiographic diagnosis of “pistol-grip-deformity” Hip Int 2011 21 6 732 739 10.5301/HIP.2011.8821 22101621

[B32] Clohisy JC Carlisle JC Trousdale R Kim YJ Beaule PE Morgan P Steger-May K Schoenecker PL Millis M Radiographic evaluation of the hip has limited reliability Clin Orthop Relat Res 2009 467 3 666 675 10.1007/s11999-008-0626-4 19048356 PMC2635468

[B33] Gosvig KK Jacobsen S Sonne-Holm S Palm H Troelsen A Prevalence of malformations of the hip joint and their relationship to sex, groin pain, and risk of osteoarthritis: a population-based survey J Bone Joint Surg Am 2010 92 5 1162 1169 20439662 10.2106/JBJS.H.01674

[B34] Bland JM Altman DG Measuring agreement in method comparison studies Stat Methods Med Res 1999 8 135 160 10.1191/096228099673819272 10501650

[B35] Bland JM Altman DG A note on the use of the intracalss correlation coefficient in the evaluation of agreement between two methods of measurement Comput Biol Med 1990 20 337 340 10.1016/0010-4825(90)90013-F 2257734

